# Gastric Electrical Dysarrhythmia in Probable Rapid Eye Movement Sleep Behavior Disorder

**DOI:** 10.3389/fneur.2021.687215

**Published:** 2021-08-26

**Authors:** Anjiao Peng, Shuming Ji, Wanling Li, Wanlin Lai, Xiangmiao Qiu, Shixu He, Bosi Dong, Cheng Huang, Lei Chen

**Affiliations:** ^1^Department of Neurology and National Clinical Research Center for Geriatrics, West China Hospital, Sichuan University, Chengdu, China; ^2^Department of Project Design and Statistics, West China Hospital, Sichuan University, Chengdu, China; ^3^Department of Rehabilitation Medicine Center, West China Hospital of Sichuan University, Chengdu, China

**Keywords:** REM sleep behavior disorder, Parkinson's disease, electrogastroenterogram, gastrointesinal disorders, neurodegenarative disease

## Abstract

**Background:** Subjective gastrointestinal complaints have been repeatedly reported in patients with REM sleep behavior disorder (RBD), but objective evidence is scarce. We aimed to objectively investigate the gastrointestinal dysfunction in individuals with probable RBD (pRBD) using an electrogastrogram.

**Methods:** Thirty-two participants with pRBD and 60 age- and gender-matched healthy controls were enrolled. pRBD was diagnosed based on questionnaires and further assessed by experienced neurologists. After thorough assessment of participants' subjective gastrointestinal symptoms, preprandial and postprandial gastric activities were measured using an electrogastrogram. Dominant frequency, dominant power ratio, and the ratio of preprandial to postprandial power were analyzed.

**Results:** Among the gastric symptoms, hiccup (34.8 vs. 9.6%, *p* = 0.017) and postprandial gastric discomfort (43.5 vs. 15.4%, *p* = 0.017) were more frequent in participants with pRBD than in controls. The dominant frequency on the electrode overlying the gastric pyloric antrum was lower in pRBD than in healthy controls (2.9 [2.6–2.9] vs. 2.9 [2.9–3.2] cpm, *p* = 0.006). A reduced dominant power ratio from the same electrode was also found in individuals with pRBD (60.7 [58.0–64.5] vs. 64.2 [58.7–69.6] %, *p* = 0.046).

**Conclusion:** Patients with pRBD have a higher rate of gastric dysfunction, which presented as irregular slow wave rhythmicity on an electrogastrogram.

## Introduction

Rapid eye movement (REM) sleep behavior disorder (RBD), also known as REM sleep without atonia, is characterized by symptoms of dream enactment and loss of muscle atonia during REM sleep (1, 2). RBD is now believed to be a prodromal marker of synucleinopathy neurodegenerative diseases such as Parkinson' s disease (PD), dementia with Lewy bodies, multiple system atrophy, and pure autonomic failure (3–6). RBD can occur years to decades before the onset of motor symptoms (4, 7).

Bidirectional regulation of the gut–brain axis is a hot spot in recent years. The central nervous system regulates gastrointestinal function through sympathetic and parasympathetic pathways. Conversely, the gastrointestinal tract could also regulate the central nervous system through a variety of ways, including vagus reflexes and metabolites of the gut microbiota (8). Gastrointestinal dysfunction was found to play an important role in the pathogenesis of PD and other neurodegenerative diseases (9). Studies also show that subjective gastrointestinal dysfunctions, such as delayed gastric emptying and constipation, were common in PD and its prodromal RBD (10, 11); however, objective evidence is still scarce.

The motility of the stomach muscle is mediated by a myogenic mechanism, which is composed of gastric slow waves and spike/second potentials at a rate of three cycles per minute. The slow wave triggers the spike potential (or action potential) and then elicits contraction of the stomach muscle (11). These waves can be measured noninvasively using cutaneous electrogastrogram (EGG) recorders placed on the abdominal skin (12, 13). EGG recording is now clinically used to evaluate gastric motility in many disorders such as functional dyspepsia (13) and diabetic gastropathy (14). Studies also show that using EGG could accurately and objectively evaluate the gastrointestinal function in patients with PD (11, 15, 16). The aim of this study was to compare the gastric rhythms between individuals with RBD and healthy controls using EGG.

## Methods

### Participants

This study was conducted with approval from the ethics committee of West China Hospital of Sichuan University (No. 2021-466), and informed consent were obtained from all participants. Probable RBD (pRBD) was diagnosed based on the validated RBD questionnaire Hong Kong (RBDQ-HK) and further assessed by neurologists from November 4, 2020, to January 21, 2021. The RBDQ-HK consists of 13 questions related to clinical features of RBD. The sensitivity (82.2–85%), specificity (81–86.9%), internal consistency, and test–retest reliability of this questionnaire in the China population have been confirmed by previous validation studies (17, 18). The total score ranges from 0 to 100, and a diagnosis of pRBD can be considered with a score of 17 or higher. After initial screening, a neurologist would further assess the symptoms and confirm the diagnosis of pRBD. Participants were excluded if they had history of the following diseases or conditions: PD; gastrointestinal diseases; abdominal surgery; diabetes; thyroid dysfunction; neuropsychiatric diseases; serious systemic disorders, such as heart, liver, or kidney failure; or had taken any medications 1 week prior to examination.

### Gastrointestinal Symptom Assessment

Before the EGG was done, the following gastrointestinal symptoms 1 week prior to examination were assessed in each participant using a predesigned questionnaire that included the following information: hiccup, bad breath, postprandial gastric discomfort, flatulence, abdominal pain, diarrhea, and constipation. The first three symptoms were used to assess the gastric function, and the rest were used to assess the enteric function.

### EGG Measurement

Gastrointestinal myoelectrical activity was measured and collected by using an EGG recorder (XDJ-S8, Hefei Kaili Co., Hefei, China). All participants were told to avoid alcohol and spicy, greasy, or irritating food for 3 days and fast for at least 6 h before the examination. The measurement was performed in a supine position. Four gastric electrodes (Hanjie Co. Ltd., Shanghai, China) were placed on the abdominal skin as follows: electrode one: 1 cm above the midpoint between umbilicus and xiphoid and 3–5 cm to the left of the midline; electrode two: the upper third quarter point from umbilicus to xiphoid; electrode three: the first quarter point from umbilicus to xiphoid; and electrode four: 2–4 cm to the right of the midpoint between umbilicus and xiphoid (19). The reference electrode was placed on the medial side of the wrist, and the grounding electrode was placed on the ipsilateral ankle. All participants were told to avoid any movement and talking during the examination. After a 6-min preprandial EGG recording, participants were instructed to take standard food containing 50 g of bread and 400 ml water in 5 min. After that, another 6-min postprandial recording was collected.

### EGG Parameters

The EGG recordings were made at sampling rate of 1 Hz and filtered with a high cutoff frequency of 0.1 Hz and a low cutoff frequency of 0.008 Hz to filter out background noise, including heartbeat. After visual inspection for artifacts, the raw EGG data was automatically calculated by the computer alongside the EGG, and the following parameters of each electrode were analyzed separately. The dominant frequency was derived from the power spectral density assessed by the periodogram method and the normal range was 2.4–3.6 cpm (19). The dominant frequency of the EGG is shown in a previous study to be equal to the frequency of the gastric slow wave measured from implanted serosal electrodes (20, 21). The dominant power was defined as the power at the dominant frequency. The dominant power ratio was defined as the ratio of dominant power to the power of the whole waveform. Considering that the position of the stomach moved largely after a meal, only preprandial data of each electrode were analyzed. To evaluate the effect of the meal on EGG, the preprandial and postprandial powers were compared, and a ratio less than one means a descent of gastric motility.

### Statistical Analysis

Normally distributed variables were presented as mean and standard deviation (SD) and calculated using unpaired *t*-tests. Nonnormally distributed data and EGG data were presented as median and interquartile range and analyzed by Mann-Whitney *U*-tests. Categorical variables were presented as frequencies and analyzed with χ^2^-tests or Fisher's exact test. Pearson correlation analysis was used to analyze the correlation between disease duration and EGG parameters. In this study, we did not perform multiple comparisons, but the exact *p*-values are all listed. All analyses were conducted using SPSS version 20 (IBM) and Prism 6 (GraphPad Software, USA). A statistical significance was considered when *P* < 0.05.

## Results

### Demographic Features

**Figure 1:**
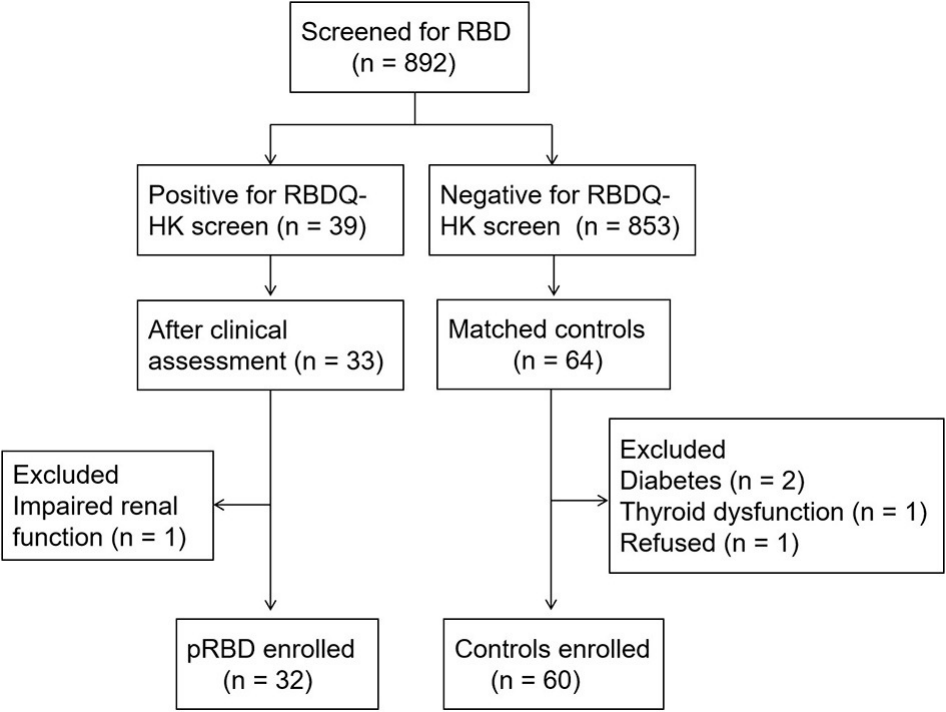
The flowchart of participant enrollment.

Of the 892 participants who had been screened by RBDQ-HK, 39 (4.37%) participants who had scores of 17 or higher received further clinical assessment. Thirty-three participants were diagnosed with pRBD, but one of them with impaired renal function was excluded. The remaining 32 participants (14 male and 18 female) were enrolled in the analyses. Another 60 age- and gender-matched participants (24 male and 36 female) who were negative for the RBDQ-HK screen were randomly selected as controls (Table 1). The flowchart is shown in Figure 1.

**Table 1:** The demographic features of controls and participants with probable RBD.

	**Probable RBD (*n* = 32)**	**Controls (*n* = 60)**	***P*-value**
Age (year, mean ± SD)	57.1 ± 9.3	56.3 ± 5.8	0.643
Gender (male, %)	14 (43.4%)	24 (40.0%)	0.825
Body mass index	24.2 ± 2.9	24.2 ± 3.1	0.939
RBDQ-HK (mean ± SD)	27.7 ± 10.9	6.3 ± 4.6	**0.000**
Duration of RBD symptoms (median, IQR)	3 [2–10]	-	-

*IQR, inter-quartile range; RBDQ-HK, REM sleep behavior disorder questionnaire Hong Kong; RBD, REM sleep behavior disorder; SD, standard deviation. Bold values means p < 0.001*.

### Gastrointestinal Function

**Figure 2:**
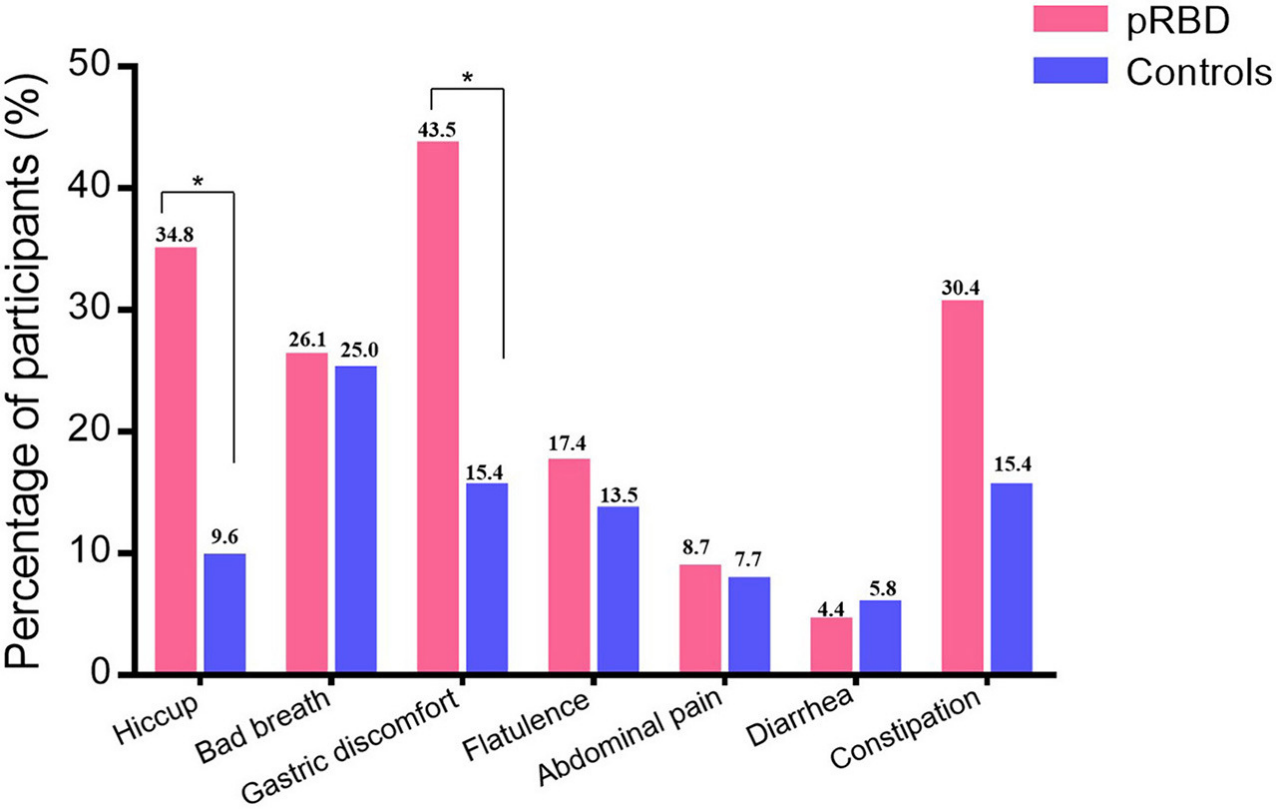
The subjective gastrointestinal function in healthy controls and participants with probable REM behavior disorder (pRBD). The prevalence of hiccup and postprandial gastric discomfort were higher in participants of pRBD than healthy controls (34.8 vs. 9.6%, *p* = 0.017; 43.5 vs. 15.4%, *p* = 0.017). The prevalence of bad breath (*p* = 0.61), flatulence (*p* = 0.73), diarrhea (*p* = 1.0), abdominal pain (*p* = 1.0), and constipation (*p* = 0.2) were similar between two groups. *P < 0.05.

The results show that the gastric symptoms of hiccup (34.8 vs. 9.6%, *p* = 0.017) and postprandial gastric discomfort (43.5 vs. 15.4%, *p* = 0.017) were more common in participants with pRBD than healthy controls. There was no significant difference between the two groups considering the prevalence of bad breath (*p* = 0.61), flatulence (*p* = 0.73), diarrhea (*p* = 1.0), abdominal pain (*p* = 1.0), and constipation (*p* = 0.2; Figure 2).

### EGG Parameters

**Figure 3:**
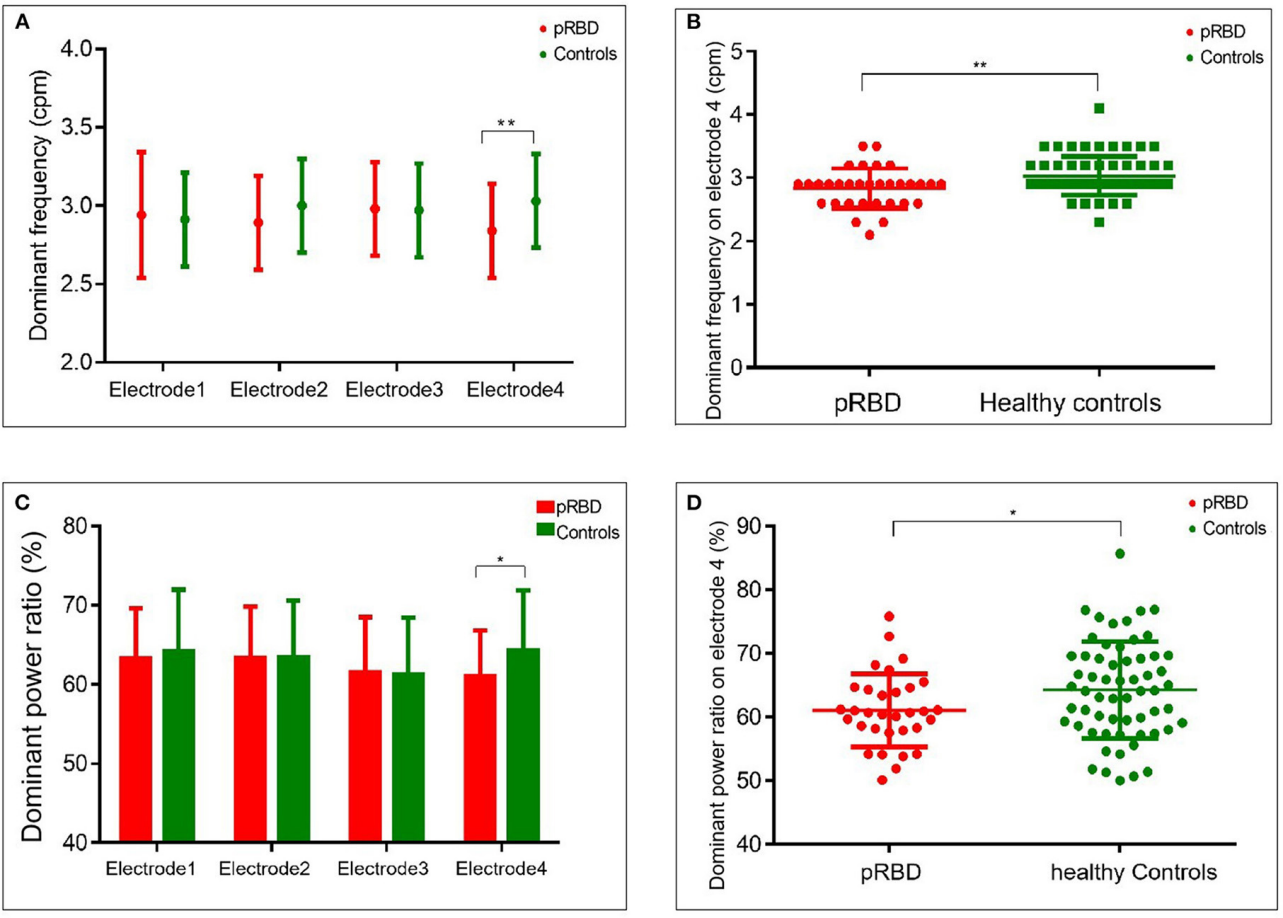
Comparison of dominant frequency and dominant power ratio between probable REM behavior disorder (pRBD) and healthy controls. The dominant frequency was lower in pRBD than that of controls on electrode four **(A,B)**. A reduced dominant power ratio from the same electrode was also found on electrode four, overlying the gastric pyloric antrum **(C,D)**. *P < 0.05; **P < 0.01.

EGG recording showed that the dominant frequency and dominant power ratio were relatively lower in participants of pRBD than in healthy controls on electrode four. In participants with pRBD, the average dominant frequency was 2.9 [2.6–2.9] cpm on electrode four, lower than that of healthy controls (2.9 [2.9–3.2] cpm, *p* = 0.006). No significant difference of dominant frequency was detected in the other electrodes (Figures 3A,B and Table 2). Besides this, reduced dominant power ratio of EGG was also detected between pRBD and healthy controls (60.7 [58.0–64.5] % vs. 64.2 [58.7–69.6], *p* = 0.046) on the electrode four (Figures 3C,D). The ratio of preprandial to postprandial power was also compared, but no significant difference was noted between pRBD and healthy controls.

**Table 2:** The electrogastrogram parameters of controls and participants with probable RBD^*^.

	**Probable RBD (*n* = 32)**	**Controls (*n* = 60)**	***P*-value**
**Body of stomach (electrode 1)**			
Dominant frequency (cpm)	2.9 [2.9–3.2]	2.9 [2.9–3.1]	0.411
Dominant power ratio (%)	61.6 [59.1–67.9]	63.6 [59.0–68.5]	0.494
Preprandial/postprandial power ratio	0.9 [0.5–1.1]	1.0 [0.6–1.4]	0.122
**Lesser curvature of stomach (electrode 2)**			
Dominant frequency (cpm)	2.9 [2.7–3.2]	2.9 [2.9–3.2]	0.185
Dominant power ratio (%)	62.9 [59.3–68.2]	61.4 [58.2–68.5]	0.682
Preprandial/postprandial power ratio	0.9 [0.6–1.3]	1.1 [0.6–1.6]	0.160
**Greater curvature of stomach (electrode 3)**			
Dominant frequency (cpm)	2.9 [2.9–3.2]	2.9 [2.9–3.2]	0.607
Dominant power ratio (%)	60.3 [57.5–64.2]	61.0 [55.0–65.6]	0.977
Preprandial/postprandial power ratio	1.1 [0.7–1.7]	1.1 [0.7–1.7]	0.815
**Antrum curvature of stomach (electrode 4)**			
Dominant frequency (cpm)	2.9 [2.6–2.9]	2.9 [2.9–3.2]	**0.006**
Dominant power ratio (%)	60.7 [58.0–64.5]	64.2 [58.7–69.6]	**0.046**
Preprandial/postprandial power ratio	1.0 [0.8–1.5]	1.1 [0.6–1.7]	0.963

* *Data were shown as median and inter-quartile range*.

*Cpm, count per minute; RBD, REM sleep behavior disorder*.

Because the dominant frequency and dominant power ratio on electrode four were altered in participants with pRBD, we further analyzed their correlation with clinical symptoms. The results show that RBD symptom duration was not statistically associated with dominant frequency (*p* = 0.784) or dominant power ratio (*p* = 0.058).

## Discussion

Results of this study show that the rate of gastric dysfunction was higher in individuals with pRBD. Using EGG, the impaired rhythmicity can be objectively measured, which was represented as decreased dominant frequency and dominant power ratio.

Upper gastrointestinal symptoms, such as nausea, dyspepsia, and abdominal fullness, were common in patients with PD and also RBD (10, 11). These symptoms represent a delayed emptying of the stomach (22, 23). In this study, we also found that gastric dysfunctions (including hiccup and postprandial gastric discomfort) were more frequent in individuals with pRBD. Gastric dysarrhythmia was further confirmed using EGG, which was reflected as reduced dominant frequency and dominant power ratio. However, previous studies show that gastric motility and the gastric transit time were normal in participants with RBD (24, 25). This discrepancy can be explained for both the 13C-octanoate breath test and gastrointestinal transit time in previous studies were used to evaluate the overall function of the stomach. However, in participants with RBD, although myoelectrical dysrhythmicity existed, the gastric dysfunction was relatively normal. In our study, we evaluated the electrical rhythm of different parts of the stomach; alteration of rhythm was only found in the gastric antrum. This topical gastric myoelectrical dysrhythmicity might not be sufficient to cause an overall change of stomach function.

Previously, the gastrointestinal dysfunction in PD was thought to be the adverse effect of treatment or the disease itself (11, 26). By using EGG, we revealed the existence of gastric dysarrhythmia in individuals with pRBD who were free from motor symptoms and corresponding treatment. Noteworthily, the reduced dominant frequency was also found in PD (11). Together, these results highlight that gastric dyspepsia in PD may be due to the disease *de novo* and could occur long before the appearance of symptoms of movement disorders.

Interestingly, the altered dominant frequency and power ratio was found on electrode four, which was thought to be above the gastric antrum rather than the proximal stomach where the pacemaker cells (i.e., ICC) were believed to be located (11, 15). To the best of our knowledge, this is the first time that different sites of myoelectrical rhythm were recorded. Given that the myoelectric rhythm of the stomach originates from the proximal stomach, passing through the gastric antrum, and then transmits to the pylorus, our results indicate that the conduction rather than the origination of gastric electrical activity is disrupted in RBD. But this hypothesis needs to be further studied using more accurate implanted serosal electrodes.

There are some limitations in this study. First of all, the diagnosis of RBD was not confirmed by polysomnography. Therefore, we could not rule out the possibility that, a relatively small number of participants might be mistakenly diagnosed with RBD. However, all participants were assessed by experienced neurologists, which could largely reduce this possibility for existing evidence showed that clinical diagnoses by neurologists provided good sensitivity (100%) and specificity (99.6%) in diagnosing RBD (27). Even if a few subjects were mistakenly diagnosed with RBD, the positive results from this study further confirm the gastric dysrhythmia in RBD. Second, the sample size is relatively small; therefore, our conclusion may not be so robust. Third, we used abdominal electrodes in this study. Although the projections of different gastric structures on the body surface are fixed in most cases, individual differences could not be excluded. However, in spite of these limitations, we recorded and confirmed gastric dysrhythmia in RBD patients for the first time.

## Conclusions

Participants with pRBD are prone to suffer from gastric dysfunction and corresponding EGG exhibits more irregular slow wave rhythmicity, indicating impaired conduction of gastric electrical activity.

## Data Availability Statement

The raw data supporting the conclusions of this article will be made available by the authors, without undue reservation.

## Ethics Statement

The studies involving human participants were reviewed and approved by the Ethics Committee of the West China Hospital of Sichuan University (No. 2021-466). The patients/participants provided their written informed consent to participate in this study.

## Author Contributions

AP designed the study, conducted the study, carried out the statistical analysis, and drafted the manuscript. SJ carried out the statistical analysis. WLi, WLai, XQ, and SH collected participants and performed the electrogastrogram. BD and CH drafted the manuscript. LC designed the study and revised the manuscript. All authors contributed to the article and approved the submitted version.

## Conflict of Interest

The authors declare that the research was conducted in the absence of any commercial or financial relationships that could be construed as a potential conflict of interest.

## Publisher's Note

All claims expressed in this article are solely those of the authors and do not necessarily represent those of their affiliated organizations, or those of the publisher, the editors and the reviewers. Any product that may be evaluated in this article, or claim that may be made by its manufacturer, is not guaranteed or endorsed by the publisher.
